# The digital era and the psychiatric follow up during COVID-19 pandemic– are we ready?

**DOI:** 10.1192/j.eurpsy.2021.782

**Published:** 2021-08-13

**Authors:** G. Andrade, M.J. Gonçalves, C. Sereijo

**Affiliations:** 1 Serviço De Psiquiatria E Saúde Mental, Centro Hospitalar Universitário Lisboa Norte, Lisboa, Portugal; 2 Serviço De Psiquiatria E Saúde Mental, Centro Hospitalar Universitario Lisboa Norte, Lisboa, Portugal

**Keywords:** COVID-19, psychiatry, health communication

## Abstract

**Introduction:**

The pandemic caused by the SARS CoV-2 virus (COVID-19) has a profound effect in the health care system (HCS). The therapeutic effect of communication skills is well known. Psychiatric patients are a vulnerable population and remote care via telephone was one of the first implemented measures during the lockdown.

**Objectives:**

The aim is to highlight the potential benefits and risks of remote follow up, according to the scientific evidence currently available.

**Methods:**

Non-systematic review of the literature with the selection of scientific articles published in the last year. The search was performed in Pubmed database with the following Mesh terms: “COVID-19”, “psychiatry”, and “health communication”. Complementary references were also included.

**Results:**

For those with a stable psychiatric condition, remote appointments may guarantee the adequate follow up in a safe way throughout the COVID-19 pandemic. However, telephonic appointments are associated with a limited ability to perform psychopathological examination. A better assessment can be achieved if video call is used. Also, data protection and the ability of giving informed consent by psychiatric patients should be addressed. Additional training should be considered. A subgroup of patients with severe mental illness may require face-to-face visits.

**Conclusions:**

COVID-19 pandemic is an unprecedented crisis and telemedicine is now emerging as an alternative. Remote consultation has advantages and, in some situations, it may replace or complement the in-person visits. Since social isolation is one of the most effective measures, digital means constitute a window of opportunity for the HCS.

